# Temporal Dynamics and Surveillance of Highly Pathogenic H5 Avian Influenza in Wild Birds in Northern Serbia (2016–2025)

**DOI:** 10.3390/vetsci12090894

**Published:** 2025-09-15

**Authors:** Biljana Djurdjević, Milena Samojlović, Diana Lupulović, Tamaš Petrović, Vladimir Polaček, Slobodan Knežević, Marko Pajić

**Affiliations:** 1 Scientific Veterinary Institute “Novi Sad”, 21000 Novi Sad, Serbia; milena.s@niv.ns.ac.rs (M.S.); tomy@niv.ns.ac.rs (T.P.); vlade@niv.ns.ac.rs (V.P.); slobodan.knezevic@niv.ns.ac.rs (S.K.); markopajic@niv.ns.ac.rs (M.P.); 2 Institute for the Application of Nuclear Energy, 11080 Belgrade, Serbia; diana.lupulovic@inep.co.rs

**Keywords:** HPAI, northern Serbia, outbreaks, surveillance, wild birds

## Abstract

Wild birds, particularly migratory waterfowl (family Anatidae), are the primary reservoir of avian influenza viruses, playing a key role in their transboundary spread. In the winter of 2016/2017, the H5N8 strain was first recorded in Serbia, with 20 outbreaks confirmed in wild birds by 2021, mostly involving mute swans in the Vojvodina region. From 2021 onward, the dominant strain was H5N1, with 38 confirmed outbreaks up to 2023, primarily affecting mute swans, black-headed gulls, and common cranes. Sporadic H5N2 outbreaks were also detected, with two cases in 2021 and 2023. Due to its rich ornithofauna, natural reserves, and habitats suitable for migratory birds, the Vojvodina region has been officially designated as a high-risk zone by the Veterinary Directorate of the Ministry of Agriculture, Forestry and Water Management of the Republic of Serbia. While sporadic cases occurred in backyard poultry, commercial farms remained unaffected. Infections peaked in autumn and early spring. Control measures included enhanced biosecurity, movement restrictions, and removal of dead birds to protect domestic poultry. Despite the circulation of avian influenza virus in wild birds, Serbia’s commercial poultry industry avoided outbreaks and major economic losses. Including outbreak data provided insight into the scale and persistence of avian influenza threats in this region.

## Simple Summary

Since 2016, the occurrence of highly pathogenic avian influenza (HPAI) virus in Serbia has become more frequent and widespread, with annual detections reported from 2021 onward, primarily in the northern regions. This area, with its rich bird population and suitable habitats for migrating birds, is considered a high-risk area for the disease occurrence. While some cases have been found in backyard poultry, commercial farms have not been affected. The virus has caused large die-offs of wild birds, particularly swans and, more recently, common cranes. The disease is most common in the colder months, and when it is detected, measures are taken to stop its spread, such as improving biosecurity on farms and removing infected birds. Despite the ongoing presence of the virus, Serbia’s poultry industry has so far avoided major economic losses. These findings are important for understanding how the disease spreads and for protecting both wildlife and the poultry industry in the future.

## 1. Introduction

Avian influenza viruses (AIVs) possess the ability to infect and replicate in a range of warm-blooded vertebrate species, primarily birds and some mammals, which presents a significant obstacle to global disease eradication initiatives [1]. Each year, multiple strains of avian influenza viruses circulate extensively within wild bird populations, which act as natural reservoirs primarily for domestic and wild avian species. Additionally, these viruses can occasionally infect accidental hosts, including terrestrial and aquatic mammals, such as humans [2].

Highly pathogenic avian influenza (HPAI) represents a significant threat to both poultry industries and wild bird populations globally. In recent decades, the increasing frequency of HPAI outbreaks among wild birds has raised concerns over virus persistence, evolution, and transmission along migratory routes [3]. Over the past three decades, the sustained and broad dissemination of HPAIV H5N1 viruses belonging to the gs/GD lineage in both domestic poultry and wild avian species has driven the development of a wide range of unique genotypes through viral reassortment events [4]. Concurrently, genetic drift driven by point mutations in the hemagglutinin (HA) gene—subjected to significant selective pressure—has resulted in the evolution of ten major genetic clades (designated 0–9) and multiple subclades [5]. To date, outbreaks caused by gs/GD lineage HPAIV H5Nx viruses have been reported in over 80 countries across Asia, Africa, Europe, and North America, warranting the use of the term “pandemic” due to their extensive geographic spread, high severity, and scale [5,6,7,8,9].

Northern Serbia (Vojvodina Province) characterized by extensive wetlands and critical stopover habitats for migratory waterfowl, plays a key role in regional avian ecology and presents a potential hotspot for virus introduction and spread. Given these developments, continuous monitoring and detailed investigation of HPAI outbreaks in Serbia are crucial for understanding virus dynamics, assessing risks to domestic poultry, and implementing effective control strategies.

Despite the recognized importance of wild birds in the ecology of HPAI, long-term surveillance data from this region have been limited. This study aims to address this gap by presenting comprehensive surveillance data collected over a ten-year period (2016–2025), highlighting patterns of virus detection, affected species, seasonal dynamics, and implemented control measures. The findings contribute to a better understanding of HPAI epidemiology in wild birds and support evidence-based approaches to disease control in high-risk areas.

## 2. Material and Methods

### 2.1. Data Sources and Surveillance Methods

Since the first incursion of H5N8 in 2016, surveillance of wild birds for avian influenza has been recognized as a priority within the Serbian disease monitoring system; however, it was only fully implemented nationwide in 2021 [10]. The data used in this study were collected through this coordinated national surveillance program, conducted by the Veterinary Directorate of Serbia in collaboration with regional veterinary institutes and local veterinary organizations. The surveillance system aimed to detect and characterize circulating H5 subtype highly pathogenic avian influenza viruses to inform timely control measures, including biosecurity enhancements and movement restrictions.

Surveillance included passive and active monitoring of wild birds, particularly migratory waterfowl, as well as domestic poultry farms in high-risk areas such as the Vojvodina region. Active surveillance is conducted twice annually (March–May and September–November) and targets multiple poultry-related settings. Sampling includes poultry at locations where trade occurs, such as markets, exhibitions, and fairs, as well as birds raised on individual household farms. Sampling efforts also extend to intensive poultry farms, where swabs are taken to assess the status of avian influenza virus within commercial production systems. Additionally, cloacal swabs or fecal samples are collected from wild birds to monitor potential viral presence in natural reservoirs.

Passive surveillance is carried out throughout the entire year and involves the examination of samples collected from poultry exhibiting clinical signs suggestive of avian influenza. It also includes the investigation of all cases of illness and mortality among wild birds, particularly waterfowl species, with an emphasis on high-risk wetland areas known to be critical for disease emergence and spread. Veterinary institutes are responsible for the implementation of avian influenza surveillance activities, including the collection of appropriate diagnostic samples in accordance with established protocols. Individuals such as bird watchers, ornithologists, hunters, or members of the general public who encounter dead or visibly ill wild birds are instructed to report and submit the findings to the competent veterinary institute. Upon receipt, the authorized veterinary institute is responsible for promptly forwarding carcasses, as well as oropharyngeal/tracheal or cloacal swab samples, to the National Reference Laboratory (NRL) for post-mortem examination and/or virological analysis. The implementation of this avian influenza surveillance system has served as a reliable tool for monitoring the presence or absence of avian influenza viruses in both domestic poultry and wild bird populations.

### 2.2. Outbreak and Case Definition

An outbreak was defined as the detection of one or more laboratory-confirmed cases of H5 subtype highly pathogenic avian influenza virus infection in birds occurring in the same geographical area and epidemiological period. The size of each outbreak was assessed based on the number of confirmed positive birds detected during the event. In wild bird populations, this was estimated from mortality events and laboratory-confirmed cases, whereas in domestic poultry, outbreak size was determined by the number of affected farms or flocks. This definition facilitated consistent reporting and comparative analysis of outbreak dynamics. Temporal analysis of avian influenza outbreak data was conducted using Microsoft Excel 365.

The case definition for avian influenza virus infection in this study was based primarily on molecular confirmation. Samples collected from wild and domestic birds exhibiting clinical signs consistent with avian influenza, or found dead during epidemiological investigations, were tested using real-time reverse transcription polymerase chain reaction (RT-PCR) to detect viral RNA. Only birds with positive RT-PCR results were classified as confirmed cases. Clinical observations and epidemiological data, such as mortality events and proximity to other confirmed cases, were used to guide sampling strategies and surveillance efforts but were not solely used to define a case without laboratory confirmation. This approach ensured high specificity and reliability of the diagnosis.

### 2.3. Molecular Analysis

Samples collected as part of this surveillance effort comprised cloacal and oropharyngeal swabs from live poultry, fecal swabs from wild birds obtained at locations characterized by high densities of wild bird populations (primarily waterfowl species), and tissue samples collected from dead birds found in the field. The presence of HPAI virus was detected using molecular diagnostic methods in tissue homogenates and swab suspensions prepared in sterile phosphate-buffered saline (PBS). Tissue samples (brain, spleen, lung) from dead birds were homogenized in PBS and centrifuged, with supernatants used for RNA extraction. Oropharyngeal and cloacal swabs were placed in sterile PBS immediately after sampling, transported to the laboratory on ice packs, and vortexed prior to RNA extraction. Total RNA was extracted using the IndiSpin Pathogen Kit (Indical Bioscience GmbH, Leipzig, Germany) according to the manufacturer’s instructions.

Avian influenza virus RNA was detected by TaqMan-based one-step RT-qPCR targeting the matrix (M) and H5 genes, using primers/probes and thermal profiles described by Spackman et al. [11], and the RNA UltraSense™ One-Step Quantitative RT-PCR System (Invitrogen, ThermoFisher Scientific, Waltham, MA, USA). N subtype was identified by RT-qPCR following Hoffman et al. [12], using the same commercial kit. Reactions were performed on a 7500 Fast Real-Time PCR System (Applied Biosystems, Foster City, CA, USA). Pathogenicity was confirmed by Sanger sequencing of the HA gene cleavage site as described by Slomka et al. [13]. PCR products were purified with the mi-Gel Extraction Kit (Metabion, Planegg, Germany) and sequenced bidirectionally on a Genetic Analyzer 3130 (Applied Biosystems) using BigDye 3.1 and the same primers. Sequences were compared to GenBank using BLAST (https://blast.ncbi.nlm.nih.gov/Blast.cgi), as previously desribed [14].

## 3. Highly Pathogenic Avian Influenza H5N8

Highly pathogenic avian influenza (HPAI) virus of subtype H5N8, belonging to clade 2.3.4.4 of the A/goose/Guangdong/1/1996 (Gs/GD) H5 lineage, has emerged as a significant global threat to both poultry and wild bird populations, demonstrating considerable potential for rapid spread and reassortment since its first major outbreaks in East Asia in the early 2010s [15]. Several distinct genetic clades and subclades have been identified within 2.3.4.4, including clades 2.3.4.4a through 2.3.4.4h, reflecting the virus’s evolutionary plasticity and capacity to adapt to different avian hosts and ecological niches [8,16].

In Europe, the first introduction of H5N8 occurred in 2014, likely mediated by migratory wild birds [17]. Subsequent epidemic waves were recorded in 2016–2017, 2020–2021, and beyond, with varying degrees of intensity and geographic spread. These outbreaks have resulted in the mass mortality of wild birds—particularly among waterfowl species such as swans, geese, and ducks—and have caused devastating losses in the poultry sector due to widespread culling, trade restrictions, and biosecurity disruptions [4,18]. The 2020–2021 epizootic was particularly severe, involving unprecedented numbers of wild bird species and leading to significant ecological consequences. The ongoing evolution of H5N8, including frequent reassortment with other avian influenza subtypes, continues to pose a risk for further adaptation and possible zoonotic events [19].

### Outbreaks of HPAI H5N8 in Northern Serbia

In 2016, during the global outbreak of HPAI H5N8 viruses of gs/GD clade 2.3.4.4b, virus spread across Asia into Europe reaching also Serbia [20]. Following confirmation of avian influenza in neighboring Hungary and other regional countries [21], the first reported mute swan mortality in November 2016 in the “Koviljsko-Petrovaradin rit” nature reserve triggered immediate comprehensive diagnostic investigations [22]. Initial RT-qPCR testing targeting the influenza A matrix and H and N genes conducted at the Scientific Veterinary Institute “Novi Sad” (NIV-NS) detected the presence of the highly pathogenic avian influenza H5N8 virus, later confirmed at the National Reference Laboratory of Veterinary Specialist Institute “Kraljevo”. Phylogenetic analyses have confirmed that the virus is classified within clade 2.3.4.4b [14].

**Figure 1:**
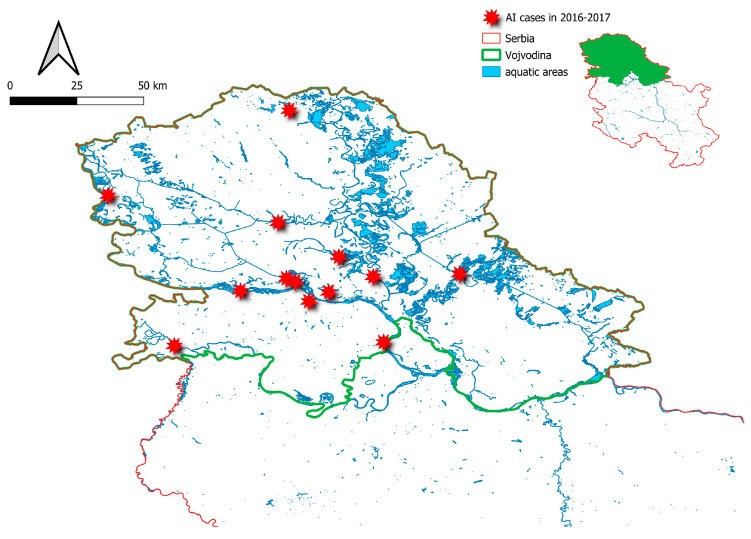
The map illustrates the spatial distribution of all confirmed HPAI H5N8 virus infection cases in wild birds across various areas in northern Serbia (Vojvodina region), from late November 2016 to the end of March 2017.

This nature reserve floodplain ecosystem along the Danube River in South Bačka District, with diverse resident and migratory bird species, provides favorable conditions for pathogen introduction and spread, with migratory birds likely playing a key role in transboundary virus transmission. After the disease emerged, protection zones and passive surveillance were established, followed by active surveillance in Vojvodina (Northern Serbia), including wild bird monitoring and sampling of domestic poultry. A total of 18 outbreaks were recorded across several districts in northern Serbia between November 2016 and March 2017, with 12 occurring in 2016 and 6 in 2017. The highest mortality rate was observed in mute swans (*Cygnus olor*) within the Koviljsko-Petrovaradin Nature Reserve. The virus was also detected in free-range domestic poultry in rural households that had direct contact with infected swans. Extensive field monitoring involved daily field surveys of the Koviljsko-Petrovaradin Nature Reserve, carried out in cooperation with the Veterinary Institute, the fisheries and forestry protection service, and park rangers. This monitoring revealed widespread infection among swans of all ages, while no other wild bird species showed signs of infection, suggesting host specificity. To control disease spread, preventive depopulation of domestic poultry was conducted in the South Bačka District, specifically in backyard holdings located in close proximity to and in contact with wild birds, resulting in the culling of 749 birds, originating from 27 households. The specialist and research staff of “NIV-NS” actively participated in outbreak management, including carcass removal and enforcing enhanced biosecurity measures on commercial poultry farms to mitigate viral incursion risks. Figure 1 illustrates the locations of HPAI H5N8 virus detections in the northern part of Serbia during the 2016–2017 season outbreaks [23].

The relatively short duration of the H5N8 avian influenza epizootic during the winter of 2016–2017 in Serbia can be partly explained by the increase in water habitat and environmental temperatures, which negatively impact the survival of the virus, as well as the reduction in the density of waterfowl populations in the spring. Compared to other European countries where the H5N8 virus was detected, the epizootic in Serbia showed limited transmission of the virus within the wild bird population.

In the following years, the highly pathogenic avian influenza virus was not detected in the Vojvodina region. Notably, during 2020, which was marked by the global COVID-19 pandemic, no cases of avian influenza were reported. This period was characterized by the implementation of strict public health measures to control the spread of the COVID-19 virus, which may have contributed to reduced avian influenza surveillance. The EFSA also indicates a marked decline in the number of detected cases of HPAI infection among wild birds in Europe during this period [24].

During 2021, isolated instances of H5N8 infection were confirmed in mute swans in the Vojvodina region [23]. Nevertheless, the number of detected cases (*n* = 2) remained substantially lower than that observed during the 2016/2017 epizootic. In the following years, no cases of infection with this HPAI subtype were recorded in the territory of Serbia. A summarized overview of all reported H5N8 virus infection cases in the northern part of Serbia, including the affected bird species, is presented in Table 1.

## 4. Highly Pathogenic Avian Influenza H5N1

The dominant H5N8 avian influenza subtype in Europe during the 2020–2021 epidemic season was largely replaced by the H5N1 subtype in the 2021–2022 epidemic season, continuing into the following period. The 2021–2022 epidemic of HPAI H5N1 is considered the largest ever recorded in the European Union, both in terms of the number of registered outbreaks in poultry industry and the geographical spread and number of wild bird fatalities [25,26]. Regarding wild birds, HPAI was predominantly detected in waterfowl (aquatic ducks), with a smaller number of cases observed in raptors and other species of wild birds [27]. During the following epidemiological year (beginning in February) especially in 2023 and 2024, HPAI H5N1 viruses spread worldwide, expanding the host range and causing mortality in multiple bird species, including mammals [28]. So far, infection with these viruses has been confirmed in more than 50 different mammal species [29]. Transmission from animals to humans has been recorded occasionally, though human-to-human transmission has not been reported. Another notable development is the loss of seasonality of the virus: according to the European Food Safety Authority (EFSA) report, the European Centre for Disease Prevention and Control (ECDC), and the European Union (EU) reference laboratory, an unprecedented number of HPAI detections were recorded in Europe during the summer months of 2021 and 2022 in both wild and domestic birds [26,30]. In previous years, a small number of cases or no cases were recorded at all during the summer period.

### Outbreaks of HPAI H5N1 in Northern Serbia

According to recent research [31], Serbia has experienced three independent incursions of the H5N1 clade 2.3.4.4b lineage since 2021, most likely originating from Central or Western European sources. From April 2021 to December 2022, a total of 16 outbreaks caused by the H5N1 virus were registered, with the outbreaks predominantly located in the Vojvodina region. During this period, swans were the most frequently affected species. However, the specificity of this epizootic is reflected in the increased number of virus detections in the population of black-headed gulls (family Laridae), resulting in significant mortality of this species, particularly in the South Bačka district. Furthermore, significant mortalities among various species of gulls were also recorded across the European continent [32]. Additionally, the Veterinary Epidemiology Unit of the Scientific Veterinary Institute “Novi Sad” actively participated in controlling the disease in the South Bačka district, where the virus was confirmed in domestic poultry on several small rural farms near the Danube–Tisa–Danube canal and the Jegrička River in the municipalities of Srbobran and Temerin. The control measures implemented during the outbreak consisted of the culling of infected poultry populations, systematic collection of cloacal swab samples, and continuous surveillance of viral presence in neighboring holdings, particularly those free-range poultry farming systems, due to their increased risk of environmental exposure to the pathogen.

In 2023, Serbia recorded 22 outbreaks of H5N1 infection in wild birds, with no cases registered in domestic poultry. Of these, 18 outbreaks were recorded in the Vojvodina region. In late autumn 2023, a significant die-off of common cranes (*Grus grus*) was documented for the first time on the European continent. The highest number of deaths was recorded in Hungary, within the Hortobágy National Park, where it was estimated that approximately 20,000 individuals of this species perished [2]. This event represents the first large-scale outbreak of H5N1 infection in cranes on the European continent, following a major die-off of cranes caused by H5N1 in Israel in 2021 [33].

A few days later, in mid-November 2023, significant common crane mortality was also reported in Serbia. In the “Slano Kopovo” Special Nature Reserve in the North Bačka district, which is an important wintering site for migratory cranes, it was estimated that over 500 individuals of this species died during this epizootic [34]. In addition to this area, sporadic cases of crane deaths were also detected in surrounding locations near Slano Kopovo. Due to their impressive size and recognizable appearance, the affected cranes were easily spotted, allowing for the registration of typical neurological symptoms, including lethargy, incoordination, and sudden deaths.

Compared to previous years, 2024 recorded the lowest number of avian influenza cases in our country, and the number of disease detections in Europe also showed some level of quiescence [35]. The improvement in the AI situation in Europe can be linked to several factors, including immunity acquired by wild birds after previous infections, a decline in populations of certain wild bird species, a reduction in environmental contamination levels, and changes in the composition of viral genotypes [35]. In 2024, Serbia recorded a total of 7 outbreaks of H5N1 AI infection, of which 3 were in the Vojvodina region (in the South Bačka and South Banat districts). Once again, the virus was detected only in wild birds (mute swans), with no reports of disease in domestic poultry. A similar epidemiological situation remained throughout 2025, with only one case of highly pathogenic avian influenza (H5N1 subtype) reported in the country as of the time of manuscript preparation, identified in mute swans in the Central Banat District during the spring season. Table 2 presents the collected data on all documented cases of H5N1 virus infection in wild birds in the northern region of Serbia since 2021 [23].

## 5. Highly Pathogenic Avian Influenza H5N2

The H5N1 Gs/GD lineage HPAI virus has undergone genetic diversification, resulting in 10 distinct groups and numerous subgroups [36]. Through reassortment with various neuraminidase subtypes, subclade 2.3.4.4 H5N1 viruses have given rise to several highly pathogenic H5 subtypes, including H5N2 [37]. Wild birds commonly harbor the H5N2 subtype of influenza virus, where it is usually of low pathogenicity. Nonetheless, following its introduction into domestic poultry populations, the virus can undergo mutations leading to the emergence of a highly pathogenic avian influenza form [38]. Since 2012, this subtype has been isolated in poultry, wild birds and mammals, including humans, pigs and dogs in Euroasia, Africa and the United States [39,40,41,42,43,44,45,46]. Although the majority of detected H5N2 virus infections have been reported in domestic poultry, causing severe outbreaks [27,45,47], research by Shearn-Bochsler et al. [48] demonstrated that this viral subtype also causes severe systemic disease in various species of raptors, indicating a high susceptibility of these wild birds to the infection. The infection pattern observed was consistent with that caused by the H5N1 virus in other avian species.

### Outbreaks of HPAI H5N2 in Northern Serbia

In Serbia, the H5N2 subtype of highly pathogenic avian influenza has been detected in only two occasions to date: first, in 2021, in tissue samples collected from deceased mallard ducks (*Anas platyrhynchos*); and subsequently, in late 2023, in a dead mute swan. Table 3 presents the collected data on all documented cases of H5N2 virus infection in wild birds in the northern region of Serbia [23].

## 6. Spatio-Temporal Distribution of HPAI Cases in Northern Serbia

**Figure 2:**
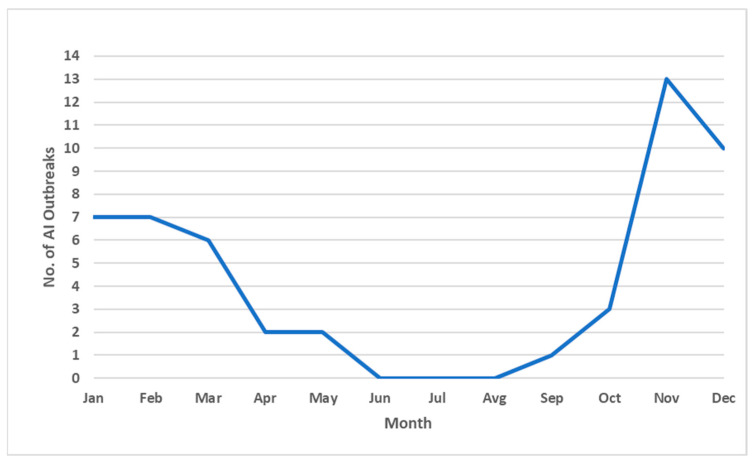
Temporal distribution of highly pathogenic avian influenza (HPAI) outbreaks by month in the Vojvodina region, covering the period from November 2016 to March 2025. The graph shows the total number of outbreaks reported per month, illustrating a clear seasonal pattern with peaks during late autumn and early winter.

All confirmed cases of infection with HPAI virus were detected exclusively during the autumn and early spring months, specifically in the period from September to the end of March (Figure 2). Notably, no cases were reported during the summer months, suggesting a marked seasonality in the occurrence of infections, potentially associated with environmental conditions or migratory patterns of wild bird populations.

**Figure 3:**
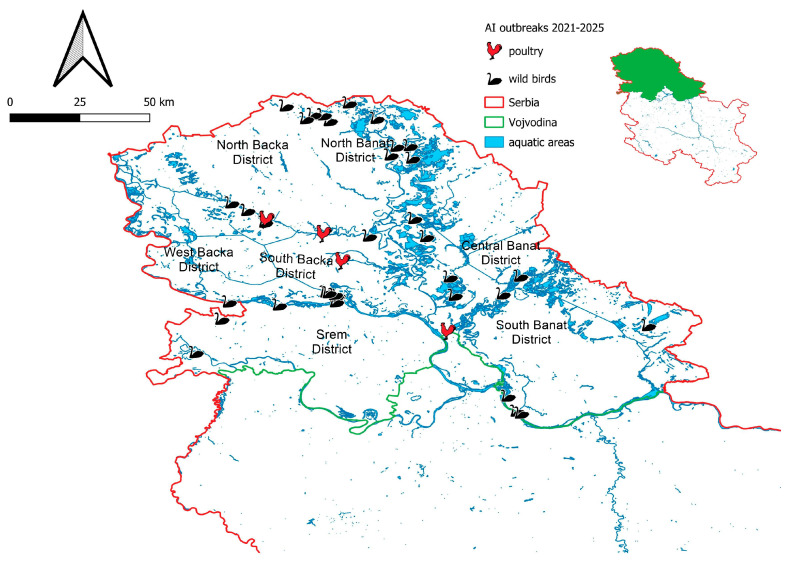
The map illustrates the spatial distribution of detected HPAI virus subtypes in wild birds across the Vojvodina region from 2021 to the present. Additionally, the figure includes recorded outbreaks in domestic poultry during the same period, providing a comparative overview of the disease dynamics in wild and domestic bird populations.

As illustrated in Figure 3, all confirmed cases of HPAI virus infection in wild birds were recorded along the banks of major rivers, including the Danube and its tributaries, as well as in nature parks, lakes, irrigation canals and ponds predominantly situated in the northern regions of Serbia, particularly in habitats commonly frequented by wild waterfowl.

## 7. Discussion

The findings presented in this study originate from a nationally coordinated surveillance system for the early detection of highly pathogenic avian influenza viruses in both domestic and wild birds. These results offer important insights into the dynamics of HPAIV circulation among wild birds in Serbia and how these findings correspond to HPAIV occurrence worldwide, and more specifically in Europe. The results of this study underscore the prominent role of waterfowl—particularly mute swans—in the maintenance, spread, and overall ecology of avian influenza in the region [49].

A substantial number of studies has documented significant mortality among swan populations associated with highly pathogenic avian influenza infections, particularly following the emergence of the H5N8 epizootic in 2014 and its subsequent replacement by or co-circulation with the globally disseminated H5N1 lineage. This trend underscores the heightened susceptibility of swans to HPAI viruses and highlights their role as both sentinel and affected species in the context of avian influenza outbreaks [50,51,52,53,54,55,56,57]. Owing to their large body size and visibility, clinical signs of disease and large-scale mortality among swans are relatively easy to detect in natural settings. A similar pattern was observed during the mass mortality events in common cranes documented in late 2023 [34]. Indeed, significant mortality can occur in highly social bird species that gather in large, dense aggregations, where pathogen transmission is facilitated and outbreaks can spread rapidly [28].

The whole territory of Vojvodina is intersected by several major migratory bird flyways, positioning this region as a critical junction within the broader European avian migratory network. Notably, the Danube Flyway, which follows the extensive river corridor and associated wetland habitats, serves as a vital stopover and staging area for numerous waterfowl and migratory bird species [58]. Additionally, the Panonian migratory route traverses the region, linking northern and southern parts of Europe, while the Adriatic–Black Sea flyway further contributes to the diversity of migratory pathways crossing this area. Due to the convergence of these migratory routes, Vojvodina experiences a high influx of diverse wild bird populations during seasonal migrations, encompassing species from various ecological groups including waterfowl, raptors, and passerines. This ecological characteristic significantly elevates the risk of introduction and dissemination of avian pathogens such as highly pathogenic avian influenza viruses. The presence of rich wetland ecosystems and abundant bird biodiversity in this region [59] creates favorable conditions for virus maintenance and transmission, thereby rendering Vojvodina a hotspot for avian influenza surveillance and outbreak potential. Consequently, understanding the dynamics of migratory bird movements through Vojvodina is essential for effective monitoring and early detection of HPAI incursions, as well as for the development of targeted control strategies aimed at mitigating the impact of avian influenza on both wild and domestic bird populations. Additionally, numerous studies have demonstrated that inland water bodies—including lakes and wetlands utilized by waterfowl—represent important risk factors for the transmission of infectious diseases within the surrounding environment. In the presence of virus-carrying waterfowl, such habitats may facilitate the introduction and spread of HPAI viruses to other potential vectors (e.g., resident avian species or small rodents), or act as sources of fomites capable of transporting the virus to nearby poultry farms [60,61,62,63].

In the Vojvodina region, the poultry industry represents a sector of significant economic and social importance, with the highest concentration of poultry farms in Serbia located within this area. Given the potential threat posed by avian influenza and other infectious diseases, the identification of optimal control strategies is crucial for mitigating economic losses and ensuring the sustainability of poultry production. Anticipating and responding effectively to future outbreaks remains a key challenge for policymakers, necessitating a comprehensive approach that includes continuous disease surveillance, biosecurity enhancement, and rapid containment measures. Among various approaches considered worldwide, vaccination has also been recognized as a valuable component of integrated disease control strategies. Vaccination against highly pathogenic avian influenza H5 viruses has been recognized as an important tool to prevent virus spread in poultry populations. Studies from China have demonstrated that vaccination programs can effectively reduce the incidence and spread of H5N8 viruses in poultry farms, thereby minimizing economic losses and limiting zoonotic risk [64]. However, vaccination should be considered as part of an integrated control strategy that includes rigorous surveillance, biosecurity measures, and rapid response to outbreaks. Challenges such as vaccine matching, virus evolution, and maintaining high vaccination coverage need to be addressed to ensure optimal efficacy.

## 8. Conclusions

Since 2021, Serbia has implemented continuous national surveillance for avian influenza, encompassing health monitoring and systematic sample collection from both commercial and backyard poultry, as well as wild bird populations. This comprehensive approach enables ongoing detection and tracking of avian influenza virus circulation within the country. Given the persistent presence of avian influenza viruses in Europe and the surrounding region, it is crucial that any unusual mortality events or suspected cases in poultry and wild birds are promptly reported to the relevant veterinary authorities. Enhanced surveillance efforts are particularly recommended during the autumn and winter months, which coincide with peak migratory periods and thus represent heightened risk for virus introduction and spread. Additionally, medium- and long-term preventive actions are vital, especially in regions with intensive poultry production such as Vojvodina, where the risk of viral exposure is elevated due to high farm density. Overall, maintaining and strengthening coordinated surveillance and control efforts are essential to mitigate the impact of highly pathogenic avian influenza on both domestic poultry industries and wild bird populations in Serbia.

## Figures and Tables

**Table 1:** Cases of HPAI H5N8 infection in wild birds in Northern Serbia.

Year	Number of Outbreaks	Affected Wild Bird Species
2016	12	Mute swan
2017	6	Mute swan, grey heron, common buzzard
2018	Not detected	/
2019	Not detected	/
2020	Not detected	/
2021	2	Mute swan

**Table 2:** Cases of HPAI H5N1 in wild birds reported in Northern Serbia.

Year	Number of Outbreaks	Affected Wild Bird Species
2021	7	Mute swan, mallard duck (wild ducks held in captivity)
2022	9	Mute swan, black-headed gulls, mallard duck
2023	18	Common crane, mute swan
2024	3	Mute swan
2025	1	Mute swan

**Table 3:** Cases of HPAI H5N2 in wild birds reported in Northern Serbia.

Year	Number of Outbreaks	Affected Wild Bird Species
2021	1	Mallard duck
2022	Not detected	/
2023	1	Mute swan

## Data Availability

The original contributions presented in this study are included in the article.
